# Protein glycosylation in lung cancer from a mass spectrometry perspective

**DOI:** 10.1002/mas.21882

**Published:** 2024-04-04

**Authors:** Mirjam Balbisi, Simon Sugár, Lilla Turiák

**Affiliations:** ^1^ MTA‐TTK Lendület (Momentum) Glycan Biomarker Research Group HUN‐REN Research Centre for Natural Sciences Budapest Hungary; ^2^ Semmelweis University Doctoral School Budapest Hungary

**Keywords:** glycosaminoglycan, lung cancer, mass spectrometry, *N*‐glycosylation, *O*‐glycosylation

## Abstract

Lung cancer is a severe disease for which better diagnostic and therapeutic approaches are urgently needed. Increasing evidence implies that aberrant protein glycosylation plays a crucial role in the pathogenesis and progression of lung cancer. Differences in glycosylation patterns have been previously observed between healthy and cancerous samples as well as between different lung cancer subtypes, which suggests untapped diagnostic potential. In addition, understanding the changes mediated by glycosylation may shed light on possible novel therapeutic targets and personalized treatment strategies for lung cancer patients. Mass spectrometry based glycomics and glycoproteomics have emerged as powerful tools for in‐depth characterization of changes in protein glycosylation, providing valuable insights into the molecular basis of lung cancer. This paper reviews the literature on the analysis of protein glycosylation in lung cancer using mass spectrometry, which is dominated by manuscripts published over the past 5 years. Studies analyzing *N*‐glycosylation, *O*‐glycosylation, and glycosaminoglycan patterns in tissue, serum, plasma, and rare biological samples of lung cancer patients are highlighted. The current knowledge on the potential utility of glycan and glycoprotein biomarkers is also discussed.

**Abbreviations**
2‐D DIGE: two‐dimensional difference gel electrophoresis
2‐D PAGE: two‐dimensional polyacrylamide gel electrophoresis
2‐DE: two‐dimensional gel electrophoresis
A1AG1: alpha‐1‐acid glycoprotein
A1BG: alpha‐1B‐glycoprotein
AC: adenocarcinoma
ACT: alpha‐1‐antichymotrypsin
ALK: anaplastic lymphoma kinase
APCS: serum amyloid p component
Asn: asparagine
AUC: area under curve
AZGP1: zinc‐alpha‐2‐glycoprotein
BAL: bronchoalveolar lavage
BLD: benign lung disease
C18: octadecyl bonded silica
C9: complement component C9
CE: capillary electrophoresis
CFB: complement factor B
CP: ceruloplasmin
CRP: C‐reactive protein
CS: chondroitin sulfate
DS: dermatan sulfate
DSIgG: disease‐specific immunoglobulin G
EGFR: epidermal growth factor receptor
ELISA: enzyme‐linked immunosorbent assay
ESI: electrospray ionization
EV: extracellular vesicle
FF: fresh‐frozen
FFPE: formalin‐fixed, paraffin‐embedded
FTICR: Fourier‐transform ion cyclotron resonance
Fuc: fucose
FUT: fucosyltransferase
Fyn: proto‐oncogene tyrosine‐protein kinase Fyn
GAG: glycosaminoglycan
Gal: galactose
GalNAc: *N*‐acetylgalactosamine
GlcNAc: *N*‐acetylglucosamine
GPI‐80: vascular noninflammatory molecule 2
HA: hyaluronan
HILIC: hydrophilic interaction chromatography
Hp: haptoglobin
HPLC: high‐performance liquid chromatography
HPX: hemopexin
HS: heparan sulfate
IGFBP3: insulin‐like growth factor‐binding protein 3
IgG: immunoglobulin G
IGHG3: immunoglobulin heavy constant gamma 3
ITGB3: integrin beta 3
iTRAQ: isobaric tags for relative and absolute quantification
KRAS: Kirsten rat sarcoma virus
LCC: large cell carcinoma
LFQ: label‐free quantification
LIT: linear ion trap
L‐PGDS: lipocalin‐type prostaglandin D synthase
LRG1: leucine‐rich alpha‐2‐glycoprotein
MALDI: matrix‐assisted laser desorption/ionization
Man: mannose
MRM: multiple reaction monitoring
MS/MS: tandem mass spectrometry
MS: mass spectrometry
NBS: 2‐nitrobenzensulfenyl
NSCLC: non‐small cell lung cancer
PAGE: polyacrylamide gel electrophoresis
PD‐1: programmed cell death protein 1
PD‐L1: programmed cell death ligand 1
PEDF: pigment epithelial differentiation factor
PG: proteoglycan
PGC: porous graphitic carbon
PNGase F: peptide‐*N*‐glycosidase F
PON1: serum paraoxonase/arylesterase 1
PTM: posttranslational modification
Q: quadrupole
QIT: quadrupole ion trap
qPCR: quantitative polymerase chain reaction
QqQ: triple quadrupole
ROC: receiver operator characteristic
SCLC: small cell lung cancer
SCX: strong cation exchange
SDS‐PAGE: sodium dodecyl sulfate‐polyacrylamide gel electrophoresis
Ser: serine
SERPINA4: kallistatin
sEV: small extracellular vesicle
SNA: sambucus nigra
SPE: solid phase extraction
SqCC: squamous cell carcinoma
Thr: threonine
TMT: tandem mass tag
TOF: time‐of‐flight
UA: uronic acid
VEGF: vascular endothelial growth factor
WGA: wheat germ agglutinin
ZIC‐HILIC: zwitterionic hydrophilic interaction chromatography

## 1 INTRODUCTION

Lung cancer affects a large number of individuals: in 2020, there were 2.2 million newly diagnosed cases and 1.8 million deaths, which represented 11.4% of cancer diagnoses and 18.0% of cancer‐related mortality (International Agency for Research on Cancer, 2023). Less than 50% of patients diagnosed with lung cancer survive more than a year after diagnosis and 80% die within 5 years (World Cancer Research Fund International, 2023).

Lung cancer can be divided into two primary types based on histology: small cell lung cancer (SCLC) and non‐small cell lung cancer (NSCLC) (Travis et al., 2015). NSCLC accounts for approximately 80‐85% (Molina et al., 2008; Suster & Mino‐Kenudson, 2020) of all lung cancer cases, making it the more prevalent form. Within NSCLC, further subdivisions exist based on histology, which include adenocarcinoma (AC), squamous cell carcinoma (SqCC), and large cell carcinoma (LCC) (Rodriguez‐Canales et al., 2016). Oncogenic driver mutations (e.g., *KRAS*, *EGFR*, or *ALK*) are also used to subset NSCLC patients, defining further subgroups and contributing to the overall diversity of lung cancer types (Grodzka et al., 2023).

Molecular targeted therapies and immunotherapies have become an integral part of clinical practice and offer significant benefits to patients (Wang et al., 2021; Wen et al., 2012). For example, if lung cancer patients have specific gene mutations, they can receive gene inhibitor‐based targeted therapy, like gefitinib, erlotinib, osimertinib for *EGFR* mutations, or crizotinib, alectinib, and brigatinib for *ALK* rearrangements (Cooper et al., 2022). A number of immune checkpoint inhibitor drugs are also approved, many of which inhibit the PD‐L1/PD‐1 interaction. Despite an initial positive response, however, patients often develop resistance to these treatments over time, accounting for a large part of cancer‐related deaths (Boumahdi & de Sauvage, 2020; Wang et al., 2019). Due to the prevalence and lethality of the disease, extensive research efforts should be directed towards a better understanding of the biology of lung cancer to improve its diagnosis and treatment.

Proteins may undergo various posttranslational modifications (PTMs), such as glycosylation, phosphorylation, acetylation, hydroxylation, and ubiquitination (Karve & Cheema, 2011). These PTMs play a key role in the regulation of the cellular environment and may therefore affect cancer pathophysiology. Glycosylation is the enzymatic addition of carbohydrate moieties (glycans) to the protein backbone, which is a highly regulated process that occurs in the endoplasmic reticulum and Golgi apparatus of eukaryotic cells (Lin et al., 2020; Stanley, 2011). According to several studies, glycosylation has a complex impact on the entire spectrum of cancer pathology (Lin & Lubman, 2024; Munkley & Elliott, 2016; Peixoto et al., 2019; Rodrigues et al., 2018). In the initiation phase, aberrant glycosylation primarily affects key proteins involved in cell growth regulation (e.g., EGFR) and contributes to uncontrolled cell growth. Altered glycosylation of adhesion molecules (e.g., integrins, cadherins) affect cell adhesion and promote invasion into surrounding tissues. Glycosylation modifications on angiogenic factors, including VEGF, play a role in angiogenesis and subsequently the nutrient supply to growing tumors. In addition, cancer cells manipulate the glycosylation of immune checkpoint proteins to avoid recognition by the immune system. During metastasis, glycosylation changes in proteins associated with cell adhesion, motility, and extracellular matrix interactions contribute to the increased migratory and invasive capabilities of cancer cells, promoting the development of secondary tumors in distant organs.

The two main types of protein glycosylation are *N*‐glycosylation and *O*‐glycosylation. *N*‐linked glycans (*N*‐glycans) are predominantly covalently attached to proteins at asparagine (Asn) residues within the consensus sequence Asn‐X‐Ser/Thr (where X can be any amino acid except proline) (Stanley et al., 2022). All eukaryotic *N*‐glycans have a common core structure of 3 mannose (Man) and 2 *N*‐acetylglucosamine (GlcNAc) units (Man_3_GlcNAc_2_Asn), and can be divided into oligomannose, complex and hybrid types. *N*‐glycosylation affects cellular processes, including protein folding, intracellular trafficking, and cell‐cell interactions (Sim et al., 2022).

*O*‐glycosylation involves the attachment of glycans to the hydroxyl oxygen atom of serine (Ser) or threonine (Thr) residues (Brockhausen et al., 2022). The most frequent *O*‐glycans are mucin‐type *O*‐glycans, which start with an *O*‐linked *N*‐acetylgalactosamine (GalNAc) unit. Unlike *N*‐glycans, *O*‐glycans do not have a well‐defined core structure; the GalNAc can be extended through four different core structures to a mature linear or branched *O*‐glycan. *O*‐glycosylation plays a role in protein stability, protein folding, cell adhesion, signal transduction, and immune response (Chia et al., 2016).

In addition to the two mentioned above, there is another class of glycoconjugates called glycosaminoglycans (GAGs). GAGs are linear polysaccharides consisting of repeating disaccharide units and are most commonly linked to proteoglycan (PG) core proteins to form proteoglycans (Pomin & Mulloy, 2018). Four classes of GAGs can be distinguished based on the disaccharide structure, of which heparan sulfate (HS, uronic acid‐*N*‐acetylglucosamine, UA‐GlcNAc units) and chondroitin/dermatan sulfate (CS/DS, uronic acid‐*N*‐acetylgalactosamine, UA‐GalNAc units) are the most widely studied (Lawrence et al., 2008; Yamada et al., 2011). During their biosynthesis, GAGs can be sulfated at multiple positions within each saccharide unit and undergo uronic acid epimerization, contributing to structural diversity. Proteoglycans are primarily found in the extracellular matrix and on the cell surface and play critical roles in cell signaling, tissue organization, and inflammation, except for serglycin, which is an intracellular proteoglycan primarily located in the granules of immune cells, and carries heparin in addition to CS (Mulloy et al., 2017).

The study of protein glycosylation can be approached from three different perspectives: (i) glycomics focuses only on the structural characterization of glycans, (ii) glycoproteomics focuses on both the structure of glycans and their localization on the protein backbone, and (iii) intact glycoproteins can also be examined revealing different proteoforms (Yang, Franc, et al., 2017). For all strategies, mass spectrometry (MS) is one of the most efficient analytical tools.

Glycomic strategies utilize enzymatic or chemical methods to release glycans (Yang, Franc, et al., 2017). Subsequently, they are typically analyzed using high‐performance liquid chromatography (HPLC) or capillary electrophoresis (CE) tandem MS (MS/MS) with electrospray ionization (ESI) or with matrix‐assisted laser desorption/ionization time‐of‐flight (MALDI‐TOF) MS (Lageveen‐Kammeijer et al., 2019; Zaia, 2010). The study of released glycan molecules has historically been challenging due to the complexity caused by the high number of structural isomers and the wide range of charges. State‐of‐the‐art MS and sample preparation techniques (e.g., derivatization, selective enzymatic digestion), however, allow rapid and efficient identification and quantification of glycans from minimal amounts of biological samples (Lageveen‐Kammeijer et al., 2019; Xie et al., 2021).

In the glycoproteomic approach, proteins are first digested into peptides using a protease such as trypsin. Then, the peptide mixture is selectively enriched for glycopeptides, and subjected to HPLC‐ESI‐MS/MS analysis. The major advantage of glycoproteomics over glycomics is the ability to obtain site‐specific information. However, this technique requires more sensitive instrumentation and careful optimization of tandem mass spectrometry parameters (Bagdonaite et al., 2022). Furthermore, software tools are more scarce, although several are constantly under development (Kawahara et al., 2021; Li et al., 2013; Tsai & Chen, 2017). This technique works well for the characterization of individual glycoproteins, but is not straightforward in the case of samples containing multiple glycoproteins (Yang, Franc, et al., 2017).

A third viable approach is the analysis of intact glycoproteins by MS, but its application to complex biological samples is limited by the large structural microheterogeneity of glycoproteins and the lack of appropriate separation methods (Reid et al., 2023).

GAG analysis typically involves the use of bacterial lyase enzymes, to produce oligo‐ or disaccharides, followed by CE or HPLC separation, and subsequent analysis by ESI‐MS in negative mode (Kubaski et al., 2017; Pepi et al., 2021). GAG disaccharides, however, are a chemically labile family of compounds covering a broad range of polarities, therefore their HPLC‐MS analysis presents a number of challenges.

In this manuscript, we aimed to collect and review the current literature on the topic of protein glycosylation in human lung cancer based on mass spectrometry studies, complementing the previously reviewed proteomic (Gasparri et al., 2020; Ling et al., 2022) and proteogenomic (Nishimura et al., 2019) studies.

**Figure 1:**
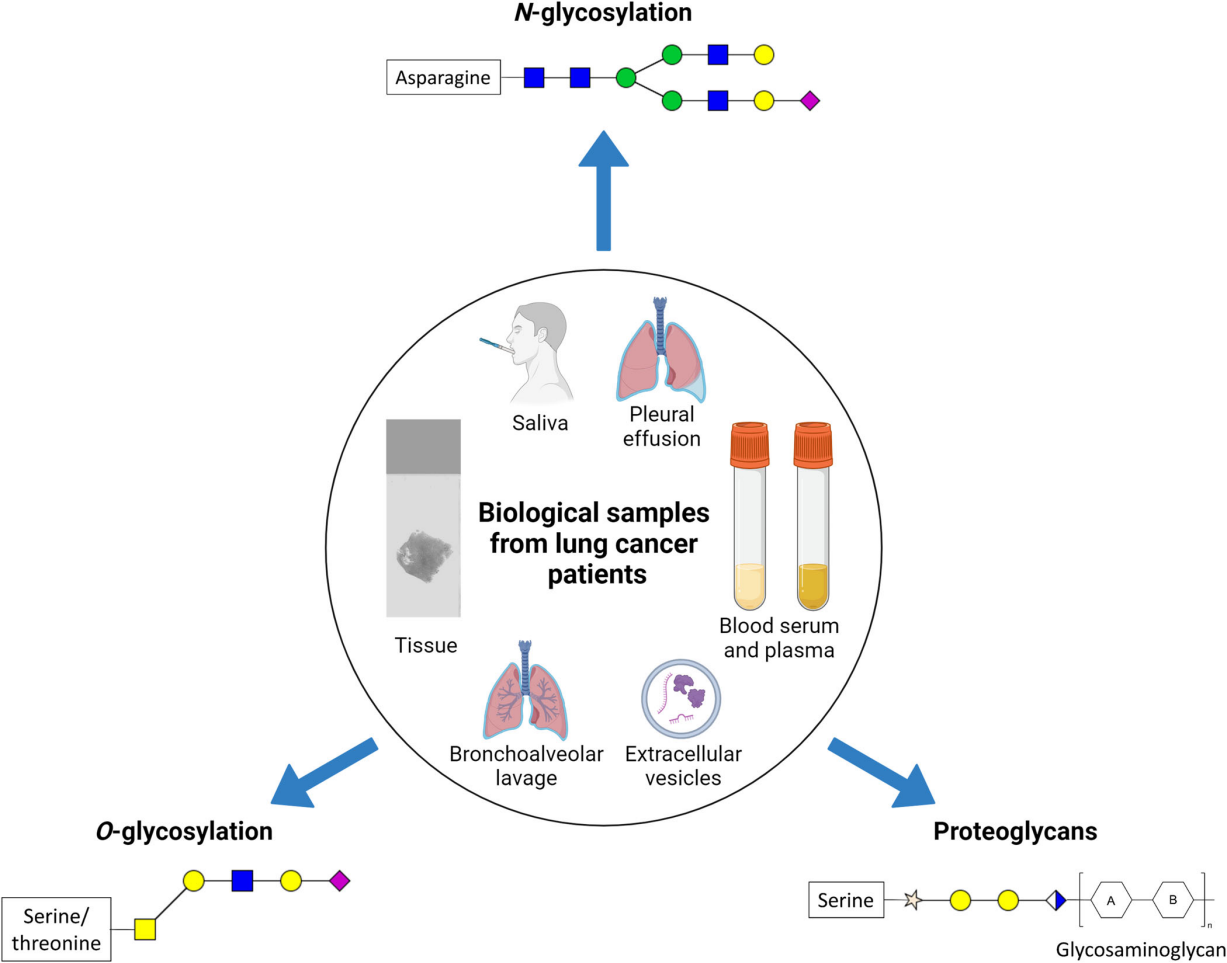
Overview of the investigated biological sample types from lung cancer patients and the potential protein glycosylation types. [Color figure can be viewed at wileyonlinelibrary.com]

To review mass spectrometry studies on protein glycosylation in lung cancer, a literature search was performed in PubMed for the term lung cancer and at least one of the following terms: glycosylation, glycan, glycopeptide, glycoprotein, glycosaminoglycan, glycomics, glycoproteomics in the title or abstract, and mass spectrometry in the text. Papers that did not fit in the scope of the review (e.g., deal with nonhuman samples or do not use mass spectrometry) were manually excluded. Early studies date back to the first decade of the 2000s, but technological advances in the meantime have made it possible to obtain more reliable information over recent years. Therefore, only papers published in 2019 or after are discussed in detail, while most important information and results of all studies are summarized in Table 1. An illustration of the biological samples and protein glycosylation types examined in the studies is shown in Figure 1.

**Table 1:** Review of all existing studies on human lung cancer samples analyzed using mass spectrometry.

Article	Sample type	Lung cancer type	Sample size for MS	Analyte	Tested protein in case of targeted study	Enzyme	Enrichment	Purification	Special sample preparation step	Type of MS instrument	Data analysis	Additional/verification techniques	Results
Glycomic, glycoproteomic, and proteomic profiling of Philippine lung cancer and peritumoral tissues: case series study of patients stages I‐III. (Alvarez et al., 2023)	FF tissue	AC	5 matched pairs	*N*‐glycans		PNGase F		PGC		Q‐TOF	MassHunter		A general increase was observed in the relative abundance of high‐mannose, fucosylated and sialofucosylated *N*‐glycans in tumor samples.
				*N*‐glycopeptides		Trypsin	HILIC			Orbitrap	Byonic, Byologic		Glycoproteins involved in key cellular processes, such as metabolism, cell adhesion, and regulatory pathways were differentially expressed.
Sialic acid linkage‐specific quantitative *N*‐glycoproteomics using selective alkylamidation and multiplex TMT‐labeling (Yang & Tian, 2022)	FF tissue		5 matched pairs	*N*‐glycopeptides		Trypsin		ZIC‐HILIC	TMT labeling, linkage‐specific sialic acid derivatization	Q‐Orbitrap	GPSeeker, GPSeekerQuan		521 differentially expressed intact *N*‐glycopeptides from 254 intact *N*‐glycoproteins were quantified. *N*‐glycoproteins sialylated at different positions are involved in different biological processes.
High‐dimensionality reduction clustering of complex carbohydrates to study lung cancer metabolic heterogeneity (Conroy et al., 2022)	FFPE tissue	AC	2	*N*‐glycans		PNGase F				Q‐TOF	Supervised learning based high‐dimensionality reduction clustering		Accurate clustering of distinct regions was observed. *N*‐glycans enriched in tumors with immune infiltration, fibrotic and necrotic regions were identified.
A new strategy for high‐efficient tandem enrichment and simultaneous profiling of *N*‐glycopeptides and phosphopeptides in lung cancer tissue (Du et al., 2022)	Tissue		1 pair, 3‐3 replicates	*N*‐glycopeptides		Trypsin	ZIC‐HILIC			Q‐Orbitrap	Byonic		1151 differentially expressed *N*‐glycopeptides and 249 differentially expressed *N*‐glycoproteins were identified.
Applicability of phenylhydrazine labeling for structural studies of fucosylated *N*‐glycans (Lattová et al., 2019)	Cells and tissue		n.i	*N*‐glycans		PNGase F, neuraminidase		Nonporous graphitized carbon	Phenylhydrazine labeling	MALDI‐TOF/TOF	Manual data evaluation		Applicability of the method was demonstrated on lung cancer samples.
Modulation of CD147‐induced matrix metalloproteinase activity: role of CD147 *N*‐glycosylation (Huang et al., 2013)	FF tissue	SqCC	1	*N*‐glycans	CD147	PNGase F	SDS‐PAGE		CD147 purification by immunoaffinity chromatography, permethylation of *N*‐glycans	LIT‐Orbitrap	Manual data evaluation	Cell biological experiments	The presence of high‐mannose and complex‐type *N*‐glycan structures in native CD147 were detected.
Glycoproteomic analysis of human lung adenocarcinomas using glycoarrays and tandem mass spectrometry: differential expression and glycosylation patterns of vimentin and fetuin A isoforms (Rho et al., 2009)	FF tissue	AC	16 matched pairs	*N*‐glycopeptides		Trypsin	Glycoproteins enriched by lectin		2‐D PAGE	LIT	SEQUEST	Glycoarray analysis on fetuin A and vimentin	8 up‐ and 7 downregulated proteins were identified.
Analysis of the human cancer glycome identifies a novel group of tumor‐associated *N*‐acetylglucosamine glycan antigens (Satomaa et al., 2009)	FFPE tissue	SCLC, AC	7 matched pairs	*N*‐glycans		No (chemical digestion)		Series of precipitation‐extraction and SPE steps		MALDI‐TOF	Manual data evaluation		5 lung cancer‐associated glycans were identified with abnormal nonreducing terminal GlcNAc residues. High‐mannose type *N*‐glycans were uniformly expressed.
Diagnostic potential of serum glycome analysis in lung cancer: a glycopattern study (Hu et al., 2024)	Blood serum	AC	16 cases at each stage (I‐IV), 16 controls	*N*‐glycans		PNGase F			Immobilization of glycoproteins, linkage‐specific sialic acid derivatization	MALDI‐TOF/TOF	GlycoWorkbench		7, mostly high‐mannose glycans were upregulated in AC, while 9 complex type glycans were downregulated. A score based on their intensities can predict the stages of cancer progression.
Intra‐individual variation in disease‐specific IgG Fc glycoform ratios to monitor the disease progression of lung cancer (Zhou et al., 2023)	Blood serum		1262 samples from 125 patients	*N*‐glycopeptides	DSIgG	Trypsin	Graphitic carbon nitride		DSIgG isolation by PAGE	MALDI‐FTICR	manual data evaluation		Measuring the ratio of 15 glycoforms might be an efficient way to monitor lung cancer progression.
Differentiation of sialyl linkages using a combination of alkyl esterification and phenylhydrazine derivatization: application for *N*‐glycan profiling in the sera of patients with lung cancer (Jezková et al., 2022)	Blood serum		39 cases, 12 controls	*N*‐glycans		PNGase F		HILIC	Esterification followed by phenylhydrazine derivatization	MALDI‐TOF/TOF	FlexAnalysis		Significant increases were observed in the amount of several tri‐ and tetraantennary glycans with mixed types of sialic acid linkages.
*N*‐glycan and glycopeptide serum biomarkers in Philippine lung cancer patients identified using liquid chromatography‐tandem mass spectrometry (Alvarez et al., 2022)	Blood serum	AC	26 cases, 22 controls	*N*‐glycans		PNGase F		PGC		Q‐TOF	MassHunter		Several highly branched sialylated and sialofucosylated *N*‐glycans were upregulated, while mono‐ and biantennary structures were downregulated in lung cancer.
				*N*‐glycopeptides		Trypsin				QqQ	MassHunter		Differentially expressed glycoproteins were mostly involved in complement and coagulation cascades, acute inflammatory response and defense response.
Disease‐specific IgG Fc glycosylation ratios as personalized biomarkers to differentiate non‐small cell lung cancer from benign lung diseases (Zhang et al., 2020)	Blood serum	NSCLC	477 cases, 509 controls	*N*‐glycopeptides	DSIgG	Trypsin	Poplar catkin enrichment		DSIgG isolation by PAGE	MALDI‐FTICR	Manual data evaluation		Higher fucosylation of DSIgG1 and DSIgG2 and lower galactosylation of DSIgG1 were observed in NSCLC compared to benign lung diseases.
Providing bionic glycome as internal standards by glycan reducing and isotope labeling for reliable and simple quantitation of *N*‐glycome based on MALDI‐ MS (Qin et al., 2019)	Blood serum		16 cases, 16 controls	*N*‐glycans		PNGase F		HILIC	Sialic acid derivatization, bionic glycome internal standard	MALDI‐QIT‐TOF	Manual data evaluation		34 *N*‐glycans were upregulated in lung cancer. 9 glycans had significant discriminatory power (AUC>0.8).
Identification a novel clinical biomarker in early diagnosis of human non‐small cell lung cancer (Jin et al., 2019)	Blood serum	NSCLC	6 cases, 6 controls (benign), 6 controls (healthy)	Deglycosylated *N*‐glycopeptides		Trypsin, PNGase F	Lectin enrichment	C18	Dimethyl labeling	Q‐TOF	ProteinPilot	PON1—Western blot, lectin‐ELISA	55 differentially expressed glycoproteins were identified.
Development of a parallel microbore hollow fiber enzyme reactor platform for online (18)O‐labeling: application to lectin‐specific lung cancer *N*‐glycoproteome. (Lee et al., 2018)	Blood serum		3 cases, 1 pooled control	Peptides derived from deglycosylated *N*‐glycoproteins		PNGase F, trypsin	Lectin enrichment		^18^O‐labeling	Q‐Orbitrap	n.i		76 peptides were quantified, of which 19 were at least 2.5‐fold up‐ or downregulated.
Microwave‐assisted deglycosylation for rapid and sensitive analysis of *N*‐glycans via glycosylamine derivatization (Wu et al., 2017)	Blood serum		16 cases, 16 controls	*N*‐glycans		PNGase F		HILIC	Glycosylamine derivatization	MALDI‐TOF/TOF	Data explorer		54 labeled glycans were detected from 50 nL of serum. Significant differences were observed in 6 glycan structures, predominantly characterized by sialylation and/or core‐fucosylation, and the two groups were completely separated based on principal component analysis.
Serum glycans as risk markers for non‐small cell lung cancer (Ruhaak et al., 2016)	Blood serum	NSCLC	Discovery set: 100 cases, test set: 108 cases + 2 controls for each case	*N*‐glycans		PNGase F		PGC		TOF	MassHunter		12 glycan variables showed significant discriminatory power (AUC>0.6), 4 were confirmed in the validation set.
Identification of GlcNAcylated alpha‐1‐antichymotrypsin as an early biomarker in human non‐small‐cell lung cancer by quantitative proteomic analysis with two lectins (Jin et al., 2016)	Blood serum	NSCLC	13 cases, 6 controls (benign), 6 controls (healthy)	peptides derived from *N*‐glycoproteins		Trypsin	Glycoproteins enriched by lectin	C18	iTRAQ labeling	Q‐TOF	ProteinPilot	ACT, A1AG1, CFB, HPX—Western blot	53 differentially expressed proteins were identified.
Integrated glycoproteomics demonstrates fucosylated serum paraoxonase 1 alterations in small cell lung cancer (Ahn et al., 2014).	Blood serum	SCLC	5 limited disease stage, 5 extensive stage, 5 control pools	peptides derived from *N*‐glycoproteins		trypsin	Fucosylated glycoproteins enriched by lectin		SDS‐PAGE, LFQ vs. iTRAQ labeling	LIT	SEQUEST, Scaffold	APCS, C9, SERPINA4, PON1—lectin ELISA, Western blot	186 proteins were identified, of which 66 were up‐ or downregulated in SCLC.
				*N*‐glycans		PNGase F		PGC	Permethylation of *N*‐glycans	MALDI‐TOF	FlexAnalysis		Increased levels of core fucosylated bi‐ and triantennary glycans were observed even in the limited disease stage.
Change in IgG1 Fc *N*‐linked glycosylation in human lung cancer: age‐ and sex‐related diagnostic potential (Chen et al., 2013)	Blood serum		Discovery set: 30 cases, 30 controls, test set: 229 cases, 380 controls	*N*‐glycopeptides	IgG	trypsin	C18		IgG separation by SDS‐PAGE	MALDI‐FTICR	Manual data evaluation		A decrease in IgG1 Fc‐galactosylation was observed in lung cancer patients, but the diagnostic ability of IgG1 Fc‐glycosylation was found to be gender‐ and age‐dependent.
Dual lectin‐based size sorting strategy to enrich targeted *N*‐glycopeptides by asymmetrical flow field‐flow fractionation: profiling lung cancer biomarkers (Kim et al., 2012)	blood serum		3 cases, 3 controls	Deglycosylated *N*‐glycopeptides		trypsin, PNGase F	Lectin complexation followed by asymmetrical flow field‐flow fractionation			LIT	Proteome Discoverer		16 up‐ and 24 downregulated *N*‐glycopeptides (with fold changes of at least 10) were identified in WGA enriched lung cancer serum, and 18 up‐ and 3 downregulated glycopeptides in case of SNA enrichment.
Glycoproteomics analysis to identify a glycoform on haptoglobin associated with lung cancer (Tsai et al., 2011)	Blood serum	AC, SqCC, SCLC, unknown type	45 cases, 26 controls	*N*‐glycans	haptoglobin	PNGase F		Extraction with chloroform	Haptoglobin separation by 2‐DE gel, permethylation of *N*‐glycans	MALDI‐TOF/TOF, LIT‐Orbitrap	MassLynx		Fucosylation level of Hp significantly increased in each subtype of lung cancer. Hp in the sera of patients with AC and SqCC displayed a higher degree of fucosylation than in the sera of patients with SCLC or unknown type of lung cancer.
Lung cancer serum biomarker discovery using glycoprotein capture and liquid chromatography mass spectrometry (Zeng et al., 2010)	Blood serum	NSCLC	9 AC, 6 SqCC, 8 control (lung disease), 8 control (healthy) pools	Deglycosylated *N*‐glycopeptides		Trypsin, PNGase F	Glycoproteins enriched by hydrazide	C18		LIT‐Orbitrap	SEQUEST	ACT, IGFBP3, L‐PGDS—ELISA	38 glycopeptides from 22 proteins were differentially expressed between at least 2 sample group, including glycoproteins involved in cell signaling and interaction, molecular transport, and cell morphology.
Glycoproteomic analysis of WGA‐bound glycoprotein biomarkers in sera from patients with lung adenocarcinoma (Hongsachart et al., 2009)	Blood serum	AC	10 cases, 10 controls	Peptides derived from *N*‐glycoproteins		Trypsin	Glycoproteins enriched by lectin	C18	Co‐immunoprecipitation, 2‐DE/2‐D DIGE	MALDI‐Q‐TOF	MASCOT, Protein Prospector	Adiponectin, ceruloplasmin, cyclin H, Fyn and GPI‐80—Western blot	27 up‐ and 12 downregulated proteins were identified.
Identification of putative serum glycoprotein biomarkers for human lung adenocarcinoma by multilectin affinity chromatography and LC‐MS/MS (Heo et al., 2007)	Blood serum	AC	3 cases, 3 controls	Peptides derived from deglycosylated *N*‐glycoproteins		PNGase F, trypsin	Glycoproteins enriched by lectin		SDS‐PAGE	LIT	Bioworks, ProtAn	Plasma kallikrein, inter‐α‐trypsin inhibitor heavy chain 3—Western blot	38 upregulated and 12 downregulated glycoproteins were identified in cancer. The majority of the upregulated proteins were associated with transport, immune responses, and inflammatory processes.
Comparative profiling of serum glycoproteome by sequential purification of glycoproteins and 2‐nitrobenzensulfenyl (NBS) stable isotope labeling: a new approach for the novel biomarker discovery for cancer (Ueda et al., 2007)	Blood serum	AC	5 cases, 5 controls	Peptides derived from *N*‐glycoproteins		Trypsin	Glycoproteins enriched by lectin		NBS labeling	MALDI‐QIT‐TOF	Mascot		34 differentially abundant serum glycoproteins were identified.
				*N*‐glycans	haptoglobin, PEDF	PNGase F		Diol‐modified monolithic silica	Immunoprecipitation, SDS‐PAGE	MALDI‐QIT‐TOF	Kompact	Lectin blot	Glycan structural changes in cancer were confirmed for haptoglobin and PEDF.
Glycoproteomics revealed novel *N*‐glycosylation biomarkers for early diagnosis of lung adenocarcinoma cancers (Fang et al., 2022)	Blood plasma	AC	20 cases, 20 controls	Deglycosylated *N*‐glycopeptides		Trypsin, PNGase F	HILIC	C18	TMT labeling	Q‐Orbitrap	Maxquant, Proteome Discoverer		17 *N*‐glycosylation sites were upregulated and 22 were downregulated in cancer. ITGB3‐680 had the highest potential for early diagnosis.
Differential proteomic approach for identification and verification of aberrantly glycosylated proteins in adenocarcinoma lung cancer (ADLC) plasmas by lectin‐capturing and targeted mass spectrometry (Ahn et al., 2014)	Blood plasma	AC	30 cases, 30 controls	*N*‐glycopeptides	A1AG1, CP	Trypsin	Glycoproteins enriched by lectin	n.i		QqQ	Manual data evaluation	ELISA	Both A1AG1 and CP were promising biomarker candidates, with AUC of 0.758 and 0.847, respectively.
Enrichment strategies in glycomics‐based lung cancer biomarker development (Ruhaak et al., 2013)	Blood plasma	AC	20 cases, 20 controls	*N*‐glycans	Untargeted + IgG	PNGase F		PGC	IgG capturing by affinity purification	TOF	MassHunter		Statistically significant differences were observed in case of four glycans, while four further glycans showed significant differences in the IgG fraction.
Ultrasensitive characterization of site‐specific glycosylation of affinity‐purified haptoglobin from lung cancer patient plasma using 10 μm i.d. porous layer open tubular liquid chromatography‐linear ion trap collision‐induced dissociation/electron transfer dissociation mass spectrometry (Wang et al., 2011)	Blood plasma	NSCLC	1 pool measured in 10 MS runs	*N*‐glycopeptides, deglycosylated *N*‐glycopeptides	Haptoglobin	Trypsin vs. trypsin, PNGase F			Immunoaffinity purification	LIT	SEQUEST		The method allowed the identification of 26 glycoforms from 10 HPLC‐MS runs using 100 fmol protein digest.
Aberrant fucosylation of saliva glycoprotein defining lung adenocarcinomas malignancy (Gao et al., 2022)	Saliva	AC	20 cases, 21 controls (other diseases), 10 controls (healthy)	*N*‐glycans		PNGase F, specific fucosidases			Linkage‐specific sialic acid derivatization	MALDI‐TOF/TOF	FlexAnalysis		The intensity of α1,2 and α1,3‐core fucosylated *N*‐glycans was significantly higher in cancer than in controls.
				deglycosylated *N*‐glycopeptides		Trypsin, PNGase F	Hydrazide			Orbitrap	n.i	Fucosyltransferases—qPCR	FUT6 and FUT11 fucosyltransferases were identified in saliva.
*N*‐glycan structures of target cancer biomarker characterized by two‐dimensional gel electrophoresis and mass spectrometry (Liu et al., 2020)	Saliva	NSCLC	10 cases, 20 controls	deglycosylated peptides derived from *N*‐glycoproteins		trypsin, PNGase F	Glycoproteins enriched by lectin			Q‐Orbitrap	Proteome Discoverer		Over 300 *N*‐glycoproteins were identified, including AZGP1.
				*N*‐glycopeptides	AZGP1	Trypsin	HILIC		2‐DE	Q‐Orbitrap	pGlyco	Western blot	5 lung cancer‐specific glycan structures have been identified.
Glycoproteomic analysis of bronchoalveolar lavage (BAL) fluid identifies tumor‐associated glycoproteins from lung adenocarcinoma (Li et al., 2013)	FFPE tissue, bronchoalveolar lavage	AC	8 BAL fluid (4 cases, 4 controls), 16 tissue (8 cases, 8 controls)	deglycosylated *N*‐glycopeptides		Trypsin, PNGase F	Hydrazide	SCX	LFQ vs. iTRAQ labeling	Orbitrap	SEQUEST, Proteome Discoverer	Napsin A—ELISA	80 glycoproteins were identified, of which 25 glycoproteins showed greater than 2‐fold difference between cancerous and benign BAL.
*N*‐glycoprotein profiling of lung adenocarcinoma pleural effusions by shotgun proteomics (Soltermann et al., 2008)	Pleural effusion	AC	5 cases, 5 controls	deglycosylated *N*‐glycopeptides		Trypsin, PNGase F	Glycoproteins enriched by hydrazide			LIT	COMET, SEQUEST, Protein‐Prophet		Several glycoproteins previously associated with tumor progression were identified in the pleural effusion.
Compositional Analysis of glycosaminoglycans in different lung cancer types—a pilot study (Pál et al., 2023)	FFPE tissue	AC, SCLC, SqCC, LCC	77 CS (41 cases, 36 controls), 76 HS (40 cases, 36 controls)	CS, HS		Chondroitinase ABC, heparinase I‐II‐III		HILIC, graphite		Q‐TOF	MassLynx		Total CS amount was significantly higher in tumor tissue than in adjacent normal tissue. CS 6‐*O*‐/4‐*O*‐sulfation differed between the lung cancer types.
Inter‐ and intratumoral proteomics and glycosaminoglycan characterization of ALK rearranged lung adenocarcinoma tissues: a pilot study (Balbisi et al., 2023)	FFPE tissue	AC	18 cases, 4 controls	CS, HS		Chondroitinase ABC, heparinase I‐II‐III		HILIC, graphite		Q‐TOF	MassLynx		Increases in both total GAG amount and average sulfation were observed in tumors. GAG‐omics profile was highly dependent on the mucin content of the region.
Glycosaminoglycans and glycolipids as potential biomarkers in lung cancer (Li et al., 2017)	FF tissue	SqCC	10 matched pairs	CS, HS, HA		Chondroitinase ABC‐ACII, heparinase I‐II‐III			HS disaccharides labeled by AMAC	QIT	n.i		Tumor samples contained more than twice as much CS as the normal ones, while no significant change was observed for the other two GAG types. The level of the 6‐sulfated CS disaccharide increased, while the level of the 4‐sulfated disaccharide decreased in cancer.
Serum proteomic profiling reveals differentially expressed IGHG3 and A1AG1 as potential predictors of chemotherapeutic response in advanced non‐small cell lung cancer (Mon et al., 2021)	Blood serum	NSCLC	14 cases (8 responders, 6 non‐responders)	peptides		Trypsin		C18		ion trap	MASCOT	IGHG3, A1AG1—Western blot	52 proteins were differentially expressed, e.g. IGHG3 was elevated, while A1AG1 was reduced in responders as compared to non‐responders.
Discovery and validation of predictive biomarkers of survival for non‐small cell lung cancer patients undergoing radical radiotherapy: two proteins with predictive value (Walker et al., 2015)	Blood plasma	SqCC	3‐3 samples from 6 cases (3: <14 month survival, 3: >18 month survival)	peptides		Trypsin		C18	iTRAQ labeling	Q‐TOF	ProteinPilot	CRP, LRG1—ELISA	658 proteins were quantified. The two groups were separated in PCA. Differentially abundant proteins included CRP and LRG1.
Glycosylated alpha‐1‐acid glycoprotein 1 as a potential lung cancer serum biomarker (Ayyub et al., 2016)	Blood serum	NSCLC, SCLC	100 cases, 50 controls	peptides		Trypsin			2‐DE	MALDI‐TOF/TOF	MASCOT, NetNGlyc	A1AG1—ELISA	8 differentially expressed proteins were identified, of which A1AG1 was validated.
Integrative proteomics and tissue microarray profiling indicate the association between overexpressed serum proteins and non‐small cell lung cancer (Liu et al., 2012)	Blood serum	NSCLC	13 cases, 5 controls	peptides		Trypsin				LIT	Bioworks, SEQUEST	A1BG, LRG1—Western blot, MRM	101 differentially expressed proteins were identified. NSCLC cases were separated from normal controls in PCA.
			70 cases, 30 controls	peptides	A1BG, LRG1	Trypsin			Internal standard peptides	QqQ	MassHunter		A1BG and LRG1 were overexpressed in NSCLC and showed AUCs of 0.816 and 0.880, respectively.
Proteomic identification of exosomal LRG1: a potential urinary biomarker for detecting NSCLC (Li et al., 2011)	Urinary exosomes	NSCLC	8 cases, 1 control pool	peptides		Trypsin			SDS‐PAGE	QIT	Spectrum Mill	Electronmicroscopy, LRG1——Western blot	18 proteins were identified. LRG1 was more abundant in NSCLC than in controls.
2‐D difference gel electrophoresis of the lung squamous cell carcinoma versus normal sera demonstrates consistent alterations in the levels of ten specific proteins (Dowling et al., 2007)	Blood serum	SqCC	8 cases, 8 controls	proteins, peptides		Trypsin		C18	2‐D DIGE	MALDI‐TOF, LIT	PMF Pro‐Found	Haptoglobin—Western blot	Several glycoproteins were differentially abundant in SqCC, including apolipoprotein A‐IV precursor, complement component C3, haptoglobin and alpha‐2‐HS glycoprotein.

## 2 *N*‐GLYCOSYLATION IN LUNG CANCER

**Figure 2:**
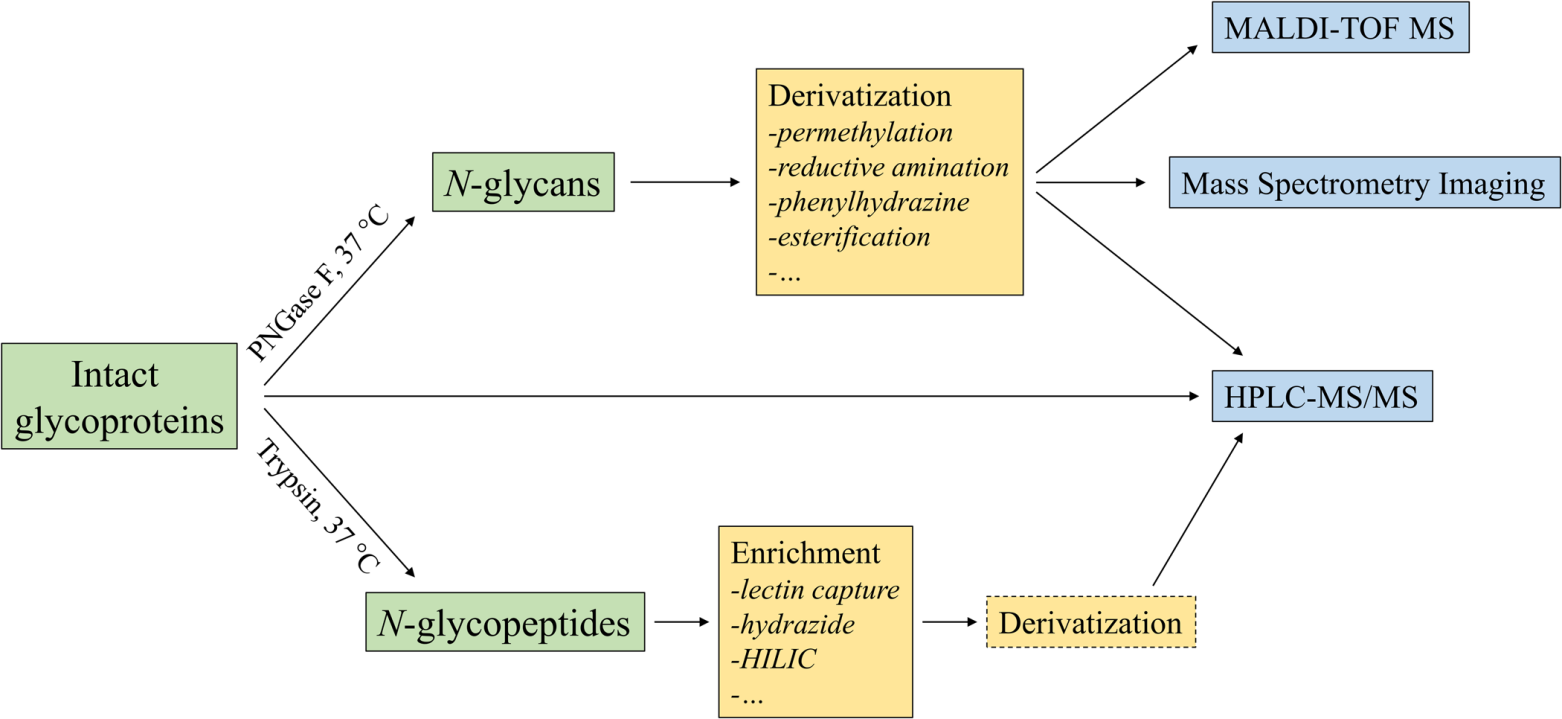
General steps of the different *N*‐glycomics workflows. The species being investigated are shown in green, the sample preparation steps in yellow and the measurement methods in blue. [Color figure can be viewed at wileyonlinelibrary.com]

Several techniques are available for sample preparation before the mass spectrometry analysis of glycosylated proteins, which are reviewed elsewhere (Illiano et al., 2020; Lageveen‐Kammeijer et al., 2022; Xiao et al., 2018). Therefore, the analytical information regarding the studies such as sample size, analyte, enzyme type, enrichment and purification mechanisms, type of mass spectrometer used and data analysis parameters are reported in Table 1 and not discussed in detail. Figure 2 provides a schematic summary of the analytical steps used in different *N*‐glycomics workflows.

### 2.1 Tissue

Mass spectrometry is suitable for the analysis of both fresh‐frozen (FF) (Yang & Tian, 2022) and formalin‐fixed, paraffin‐embedded (FFPE) (Satomaa et al., 2009; Wang et al., 2018) tissues. In the case of FFPE tissues, following deparaffinization antigen retrieval is performed to eliminate formalin‐induced crosslinks (Addis et al., 2009; O'Rourke & Padula, 2016). Tissue analysis using HPLC‐MS can be conducted in two different ways: either through tissue homogenization followed by in‐solution digestion (Alvarez et al., 2023; Du et al., 2022; Huang et al., 2013), or by performing on‐surface digestion. In the latter case, MALDI mass spectrometry imaging techniques on tissue sections can also be used (Conroy et al., 2022). Due to tumor heterogeneity, mass spectrometry imaging experiments offer additional insight for released glycans compared to pooled mass spectrometry workflows (Conroy et al., 2022). However, the MS imaging of glycopeptides is not yet feasible due to the lack of a sufficiently sensitive technique or a spatially resolved enrichment method.

In a recent study by Alvarez et al., a comprehensive investigation involving glycomic, glycoproteomic, and proteomic analyses was conducted on tissue samples of adenocarcinoma (AC) patients (Alvarez et al., 2023). A notable increase was observed in the relative abundance of high‐mannose, fucosylated and sialofucosylated *N*‐glycans within the tumor samples. Differentially expressed glycoproteins indicated the dysregulation of essential cellular processes, such as metabolism, cell adhesion, and regulatory pathways, while in case of proteins, one of the dysregulated processes was *N*‐linked glycosylation, further highlighting the relevance of the study of protein glycosylation in lung cancer research.

Similar results have been observed by others in glycoproteomic studies of lung tissue. Using a strategy for tandem enrichment of *N*‐glycopeptides and phosphopeptides, Du et al. identified 1151 differentially abundant *N*‐glycopeptides and 249 dysregulated *N*‐glycoproteins (Du et al., 2022). Gene Ontology enrichment analysis revealed that cell adhesion, extracellular matrix organization and neutrophil degranulation were the top enriched biological processes. Yang et al. investigated the functional roles of glycoproteins associated with the 303 sialylated *N*‐glycopeptides quantified using an in‐house developed linkage‐specific derivatization method (Yang & Tian, 2022). *N*‐glycoproteins with α2,6‐sialylation were found to be mainly involved in epithelial cell differentiation, cell morphogenesis, immune response and myeloid cell apoptotic process, while *N*‐glycoproteins with α2,3‐sialylation were primarily associated with cell junction organization and cell adhesion.

Conroy et al. presented MALDI mass spectrometry imaging of *N*‐glycans from AC tumor tissue (Conroy et al., 2022). Using dimensionality reduction and clustering analysis, an accurate clustering of distinct histopathological regions was observed and specific *N*‐glycans were found to be enriched in tumors with immune infiltration, fibrotic and necrotic regions. For example, the core‐fucosylated Man_3_Gal_2_GlcNAc_4_Fuc *N*‐glycan was more abundant in the fibrotic regions, while tumor infiltration resulted in increased levels of Man_9_GlcNAc_2_.

### 2.2 Blood serum

Blood serum is the medium of the blood without blood cells and clotting factors. Its analysis presents many challenges due to the large dynamic range and complexity of its components (Pietrowska et al., 2019). Albumin is the most abundant protein in blood serum, followed by globulins. Since disease specific biomarkers are typically low abundance proteins, removal of the most abundant proteins by depletion is essential (Ueda et al., 2007; Zeng et al., 2010).

The ***N*** **‐glycan** profile of blood serum from patients with lung cancer and the use of *N*‐glycans as potential biomarkers have been studied in detail in the past. Recently, Jezková et al. compared the *N*‐glycan profiles of 39 male lung cancer patients and 12 cancer‐free men using an alkyl esterification‐phenylhydrazine labeling method, which was able to discriminate 2,6‐ and 2,3‐sialic acid linkages (Jezková et al., 2022). Significant increases were observed in the levels of several tri‐ and tetraantennary glycans with mixed types of sialic acid linkages, in partial agreement with previous observations. Alvarez et al. compared the serum *N*‐glycan profiles of AC and healthy patients (Alvarez et al., 2022). The differentially abundant *N*‐glycans were found to be predominantly fucosylated, sialylated, or sialofucosylated. Several highly branched sialylated and sialofucosylated structures were overexpressed in cancer serum, while mono‐ and biantennary structures showed a significant decrease. Recently, Hu et al. compared the *N*‐glycan profiles of healthy individuals and AC patients at stage I–IV, and observed the upregulation of 7, mostly high‐mannose glycans in AC, while 9 complex type *N*‐glycans were downregulated (Hu et al., 2024). By combining information of these glycans, a score was generated which might be able to predict the AC stage, with area under curve >0.7 for most stage comparisons.

The studies mentioned above suggest that monitoring molecular changes at the glycan level may have diagnostic value and thus their applicability has been investigated. Using a Bionic glycome internal standard produced by reducing and deuterium isotope labeling of *N*‐glycans from pooled samples and the sialic acid derivatization method developed by Reiding et al. (Reiding et al., 2014), Qin et al. performed the absolute quantification of serum samples from 16 lung cancer patients and 16 healthy patients to detect glycan markers (Qin et al., 2019). In lung cancer serum, 34 *N*‐glycans were found to be upregulated. The amounts of non‐galactosylated glycans with fucose, high‐mannose glycans, and highly sialylated glycans significantly increased in lung cancer serum. Based on receiver operator characteristic (ROC) calculations, nine glycan variables, including eight glycans with α2,6‐linked sialylation, had significant discriminatory power (area under curve > 0.8) to distinguish cancer patients from controls.

A great deal of effort is directed towards exploring glycosylation changes not only at the glycan, but also at the **glycoprotein** level in lung cancer serum. Alvarez et al. aimed to identify potential *N*‐glycoprotein biomarkers from the sera of AC patients and healthy individuals (Alvarez et al., 2022). Glycopeptides corresponding to serotransferrin, alpha‐1‐antitrypsin, complement C3, and immunoglobulin M exhibited overexpression, while others corresponding to haptoglobin, complement component C8 beta chain, ceruloplasmin, alpha‐1‐antichymotrpysin, and immunoglobulin G2 showed underexpression. These glycoproteins were mostly involved in biological processes such as complement and coagulation cascades, acute inflammatory response and defense response.

Jin et al. identified a total of 55 differentially expressed glycoproteins from NSCLC serum samples, and among these, serum paraoxonase/arylesterase 1 (PON1) protein was validated by Western blot and enzyme‐linked immunosorbent assay (ELISA) (Jin et al., 2019). Combining PON1 with the previously identified glycoprotein alpha‐1‐antichymotrypsin (Jin et al., 2016) as a potential biomarker, a sensitivity and specificity of over 90% was observed. PON1 has also been proposed as a biomarker in SCLC, as Ahn et al. found an increased fucosylation of PON1 in the serum of SCLC patients (Ahn et al., 2014).

In addition to studying the glycosylation of the overall protein set, the study of glycosylation of individual proteins can also provide useful information and may be of diagnostic or therapeutic interest. Immunoglobulin G (IgG) is one of the most abundant serum proteins, containing a single glycosylation site at Asn‐297 of both heavy chains in the Fc region. The functions of IgG rely on the interaction of the Fc region with other proteins, thus Fc glycosylation has a major impact on the bioactivity of IgG (Arnold et al., 2007). To eliminate interindividual variability, Zhou et al. monitored changes during lung cancer progression (Zhou et al., 2023). Disease‐specific IgG (DSIgG) was analyzed by isolating serum immunoinflammation‐related protein complexes and it was found that measuring the ratio of 15 glycoforms might be an efficient way to monitor disease progression. In addition, Zhang et al. tested DSIgG from 509 patients with benign lung diseases (BLD) and 477 with NSCLC, and observed higher fucosylation of both DSIgG_1_ and DSIgG_2_ and lower galactosylation of DSIgG_1_ in NSCLC compared to BLD (Zhang et al., 2020).

### 2.3 Other sources

Blood plasma is less frequently used in cancer research compared to serum, but its analysis poses similar challenges. Recently, Fang et al. investigated the plasma of 20 stage I AC patients and 20 healthy controls at the glycopeptide level (Fang et al., 2022). A total of 39 *N*‐glycosylation sites were differentially occupied in AC, of which 17 and 22 were up‐ and downregulated. A site of integrin beta 3 protein (ITGB3‐680) was downregulated in AC and showed the highest potential for early diagnosis.

Although the most commonly tested biological samples are tissue and blood, saliva (Gao et al., 2022; Liu et al., 2020), pleural effusion (Soltermann et al., 2008) and bronchoalveolar lavage (Li et al., 2013) fluid can also be promising sources of lung cancer biomarkers.

As saliva is a noninvasive body fluid, its use for diagnostic purposes would be desirable. Liu et al. applied lectin affinity chromatography to analyze the salivary *N*‐glycoproteome and identified over 300 *N*‐glycoproteins (Liu et al., 2020). In an earlier study by Xiao et al., salivary zinc‐alpha‐2‐glycoprotein (AZGP1) was identified as a potential lung cancer biomarker (Xiao et al., 2012). Therefore, the glycosylation of AZGP1 was investigated as well following two‐dimensional gel electrophoresis separation. A total of 22 glycan structures were characterized, of which 5 were found to be characteristic of lung cancer. Gao et al. aimed to explore whether the fucosylation of saliva glycoproteins discriminates AC, other noncancerous diseases and healthy individuals (Gao et al., 2022). Using PNGase F and specific fucosidase enzymes, an increase in α1,2, α1,3, and α1,6‐core fucosylation was observed in AC patients compared to healthy and noncancerous patients, highlighting their diagnostic potential.

## 3 *O*‐GLYCOSYLATION IN LUNG CANCER

The study of *O*‐glycan profiles is greatly limited primarily by the lack of enzymes capable of efficiently releasing all *O*‐glycans, resulting in difficult and time‐consuming analyses (Wilkinson & Saldova, 2020). Although far less literature sources are available about lung cancer *O*‐glycosylation than *N*‐glycosylation, *O*‐glycosylation changes and their potential diagnostic applications have been investigated in several other cancer types, including colorectal cancer (Takakura et al., 2023), ovarian cancer (An et al., 2006; Yang, Höti, et al., 2017) and breast cancer (Kirmiz et al., 2007).

An overview of the significance of mucin‐type *O*‐glycans in cancer (Zhang et al., 2022) and in other human diseases (Magalhães et al., 2021) can be found in recent literature. In short, while healthy cells have mature linear or branched *O*‐GalNAc glycans, cancer cells underexpress certain glycosyltransferases, and therefore truncated *O*‐GalNAc glycans containing a single *O*‐GalNAc (Tn antigen) or a sialylated *O*‐GalNAc (sTn) are prevalent (Doud & Yeh, 2023). High expression of Tn and sTn antigens have been observed in 10‐90% of samples in several cancer types, including lung cancer (Zhang et al., 2022). Overexpression of the Tn antigen is associated with metastasis formation, while overexpression of the sTn antigen is associated with tumorigenesis, and both are related to poor prognosis. The aberrant mucin‐type *O*‐glycosylation detected in cancer is generally accompanied by the differential expression of mucins.

As there is still a lack of mass spectrometry studies on the *O*‐glycosylation of lung cancer, we present some studies using other techniques. In a study by Liang et al., serum *N*‐ and *O*‐glycan profiles in AC and squamous cell carcinoma (SqCC) patients at various stages and healthy controls were analyzed using lectin microarray analysis (Liang et al., 2019). Increased levels of T (bearing *O*‐GalNAC‐Gal units) and Tn antigens in the serum of AC patients were observed, while lower intensities were detected in SqCC patients compared to healthy controls. Similarly, López‐Ferrer et al. detected T and Tn antigens more often in AC than in SqCC by immunohistochemical staining (López‐Ferrer et al., 2002). Therefore, these antigens may be potential markers for distinguishing NSCLC subtypes. However, the study by Liang et al. also reported that serum levels of T antigen were significantly elevated only in stage III and IV AC patients, suggesting that, similar to Tn and sTn antigens, they may be related to the poor prognosis of cancer.

## 4 PROTEOGLYCANS IN LUNG CANCER

Similar to *O*‐glycosylation, there is a lack of studies on GAGs. Recently, two papers on the GAG analysis of lung cancer tissue have been published. First, the CS and HS characteristics of tumor and tumor‐adjacent normal regions from AC, SqCC, LCC and SCLC patients were studied (Pál et al., 2023). A total of 77 CS and 76 HS samples were analyzed by on surface digestion using lyase enzymes. Statistically significant changes were identified only for chondroitin sulfates: the total CS disaccharide amount was higher in tumor compared to adjacent normal samples; the abundance of the non‐sulfated component (D0a0) decreased, while the amount of the monosulfated components (D0a4 and D0a6) increased in tumor samples, in line with an earlier study (Li et al., 2017). Comparing different tumor types, the CS 6‐*O*/4‐*O*‐sulfation ratio increased significantly in AC compared to the other tumor groups. Focusing on a specific gene mutation, 22 regions from 7 *ALK*‐rearranged AC tissues were investigated in detail and increased expression levels and sulfation of CS and HS chains were observed in tumors compared to adjacent normal regions (Balbisi et al., 2023). Tumor regions were characterized by three properties: morphological classification, mucin, and stromal content. Out of the three, classification based on mucin content resulted in complete separation in principal component analysis and hierarchical clustering, indicating a strong relation between the mucin and GAG content of the tissue region.

As the aforementioned studies suggest, proteoglycans may play an important role in lung cancer. Understanding this is aided by experiments on cell cultures, and MS is a useful tool for this purpose. Serglycin was found to be frequently overexpressed in AC and it has been shown to promote NSCLC cell migration and invasion in a CD44 dependent manner (Guo et al., 2017). To gain insight into the underlying molecular mechanism, Guo et al. performed NSCLC cell culture experiments monitored by HPLC‐MS (Guo et al., 2020). The authors found that the CS‐GAG part of serglycin is the structural element that binds to the tumor cell surface CD44, thereby promoting cell migration. However, when the CS chain is removed, serglycin does not interact with CD44, which prevents cell migration. Another area of interest is the use of GAGs and their derivatives for inhibiting lung cancer. Hyaluronan tetrasaccharide has previously been used to inhibit triple‐negative breast cancer (Han et al., 2019), therefore, He et al. investigated the antitumor effects of hyaluronan tetrasaccharide derivatives on A549 AC cells (He et al., 2022). Hyaluronan tetrasaccharide derivatives containing up to 6 sulfate groups have been prepared, and those with moderate sulfation (2 or 3 sulfate units) reduced cell viability the most in vitro, and showed stronger antitumor activity in mice.

## 5 GLYCOPROTEINS INVOLVED IN LUNG CANCER ACCORDING TO PROTEOMIC STUDIES

In addition to the *N*‐/*O*‐glyco(proteo)mic and glycosaminoglycan studies discussed, several proteomic studies identified glycoproteins as potential lung cancer biomarkers. Extracellular vesicles (EVs) are lipid‐bound particles released from cells that play a pivotal role in cell signaling. As EVs reflect the physiological or pathological state of the cell from which they originate, they can be a useful source of biomarkers. Research focusing on EVs has grown substantially in the last decade (Couch et al., 2021; Mallia et al., 2020). Early proteomic studies revealed among others altered levels of haptoglobin, alpha‐2‐HS glycoprotein, alpha‐1‐acid glycoprotein 1, protein leucine‐rich alpha‐2‐glycoprotein and alpha‐1B‐glycoprotein, in serum, tissue or urinary exosome (small EV) samples of NSCLC patients, which may be potential diagnostic biomarkers (Ayyub et al., 2016; Dowling et al., 2007; Li et al., 2011; Liu et al., 2012).

In addition to diagnostic biomarkers, there are efforts to identify predictive biomarkers that can be used to predict the outcome of therapeutic interventions. It has previously been shown that the combination of C‐reactive protein and leucine‐rich alpha‐2‐glycoprotein proteins from blood plasma can predict longer survival (>18 months) after 1 week of radiotherapy (Walker et al., 2015). Later, Mon et al. identified differentially expressed proteins in the serum of patients with advanced NSCLC who responded to carboplatin plus paclitaxel chemotherapy compared to non‐responders (Mon et al., 2021). Immunoglobulin heavy constant gamma 3 was elevated, while alpha‐1‐acid glycoprotein was decreased in responders as compared to non‐responders.

Although the studies presented illustrate the role of glycoproteins in cancer and their potential use as biomarkers, most biomarker studies are in the discovery phase, requiring validation in large cohorts and a rigorous approval process for their application in clinical practice (Davis et al., 2020). As a result, the number of glycoprotein biomarkers utilized in clinical practice for lung cancer diagnosis is very limited, for example, cancer antigen 15‐3 and carcinoembryonic antigen, but these are not lung cancer specific either (Chen et al., 2023; Lin & Lubman, 2024; Nath & Mukherjee, 2014).

## 6 CONCLUSIONS AND LIMITATIONS

In this article, we reviewed the literature on the analysis of protein glycosylation in human lung cancer by mass spectrometry.

The majority of these studies focus on *N*‐glycosylation. In tissue, increases in high‐mannose glycans and terminal GlcNAc containing structures have been reported most frequently, whereas in serum and plasma, the amount of highly branched, sialylated glycans often increased, while the amount of biantennary glycans usually decreased. In glycoproteomics, most of the differentially expressed glycoproteins are extracellular or membrane proteins, which are mainly involved in cell adhesion, metabolism and immune response. There are glycoproteins whose altered expression and/or glycosylation pattern may be of diagnostic value, the most commonly studied ones being immunoglobulins, haptoglobin and CD proteins. Although the number of studies directed towards lung cancer *O*‐glycosylation and glycosaminoglycan characterization is very limited, it is clear that changes occur in both classes, mainly through increased levels of truncated *O*‐glycans and increased GAG sulfation.

Mass spectrometry detection of protein glycosylation in lung cancer is rapidly evolving due to new sample preparation strategies and the widespread use of increasingly sophisticated mass spectrometers, with Orbitraps most frequently used. However, the measurement results are limited by several factors, for example, many studies use routine proteomic collision energies in glycoproteomics, which highlights the need for careful consideration of analytical methods. In addition, different software and data analysis techniques are constantly being developed, which have improved the identification and quantification processes. Continued technical advances are expected in the future, which could lead towards more accurate, reliable and biologically meaningful insights in the field.

Mass spectrometry‐based studies to date have demonstrated that *N*‐glycosylation, *O*‐glycosylation, and glycosaminoglycan patterns of proteins are all altered in lung cancer, but the biological and clinical implications of these changes are yet to be thoroughly understood for a number of reasons. First, many studies have analyzed quite small sample sizes (see Table 1), limiting the reliability and generalizability of the results. Larger cohorts and further replication studies are necessary to establish robust associations between glycosylation changes and lung cancer, especially for specific subtypes or stages. Second, pre‐analytical factors such as cohort assembly, sample collection and storage need to be carefully considered and described in detail, which was not the case in all previous studies. Furthermore, to gain a comprehensive understanding of the disease and to develop generally applicable diagnostic and therapeutic strategies, studies across a broad spectrum of ethnicities are needed, enabling the interpretation of genetic and environmental variability.

In addition, the reliability of the biological conclusions of mass spectrometry‐based studies is closely related to analytical factors. The choice and efficiency of enrichment and purification methods, as well as the type and settings of mass spectrometers have a great impact on the accuracy and reliability of the data obtained. In addition, software used for data analysis and statistical approaches require careful consideration. Therefore, detailed documentation of sample preparation, measurement and evaluation is important to ensure reproducibility.

Last, although the studies provide insights into altered biological processes and potential biomarkers, the clinical translation of these results as reliable diagnostic or therapeutic tools is limited by the fact that many of the observed changes are related to the immune response rather than being specific to lung cancer, and especially to lung cancer subtypes. Therefore, moving towards clinical application would require rigorous validation in large and diverse patient populations using mass spectrometry and other analytical techniques.

In conclusion, the exploration of the role of protein glycosylation in lung cancer is still in the early stages, where further *N*‐glycosylation, *O*‐glycosylation and glycosaminoglycan analyses dealing with larger sample sizes are needed to enhance our understanding of biology, and to take steps towards clinical application.

## AUTHOR CONTRIBUTIONS

**Mirjam Balbisi**: Conceptualization; investigation; writing—original draft. **Simon Sugár**: Investigation; writing—review and editing. **Lilla Turiák**: Conceptualization; funding acquisition; investigation; supervision; writing—review and editing.

## CONFLICT OF INTEREST STATEMENT

The authors declare no conflict of interest.
